# Adolescence*Read at meeting of Northwest Texas Medical Association, Fort Worth, Texas, October 13, 1913.

**Published:** 1913-12

**Authors:** Alf Irby

**Affiliations:** Weatherford, Texas


THE
### TEXAS MEDICAL JOURNAL.
Established July, 1885

F. E. DANIEL, M. D., - - - - - Editor, Publisher and Proprieto

Published Monthly.—Subscription, $1.00 a Year.

Vol. XXIX. AUSTIN, DECEMBER, 1913. No. 6

The publisher is not responsible for the views of the contributors.

For Texas Medical Journal.

### Original Articles.

### Adolescence.*

BY ALF IRBY, M. D., WEATHERFORD, TEXAS.

In this eugenic crusade, when the medical profession at large is advocating vasectomy, or ex-section of the vas deferens, it seems to me that the general practitioner should feel more keenly his responsibility in relation to adolescence. This period usually extends from 14 to 25 years in the male and in the female from 12 to 21 years.

We seem to have delegated the victims of adolescence to the specialist, whereas, if the general practitioner would take a more paternal course with the youth under his care at this formative period of life, there would be little need or demand for a specialist.

The evil results of the lack of care and attention on our part are often brought first to our notice when some youth falls into the hands of the law or the alienist. This *ought not* to be.

Very few of the youths who go astray are criminals or degenerates, and it behooves us as a profession to become teachers as well as physicians.

The period of adolescence is one of evolution. Up to this time, the child is only a passive being so to speak, but now he begins a new development. The *senses*—touch, taste, sight, smell, hearing, the voice—all undergo a change. It is a period of general craving for sense-stimuli, and the many dangers and evil tendencies arising therefrom ought to make us most solicitous. In reality this is

*Read at meeting of Northwest Texas Medical Association, Fort Worth, Texas, October 13, 1913.

a period of juvenile faults, abnormalities and immoralities—an overdevelopment of the physical and sexual at the expense of the mental.

Only the physician can realize the weakness and inability of the adolescent to meet and combat the many and varied dangers which he daily and hourly encounters. He is not helped by his associates, for they necessarily of his own age and habits, making them all victims of association, and the same environments. So it devolves upon the medical profession to come to the rescue of the unfortunate youth in this most important period of life, for only we can really understand and fully appreciate the necessity for prophylaxis rather than curative remedies. It should be the purpose of every medical practitioner, when he is engaged as a physician to a family, to establish such relations with the parents of that family, that he may teach them how to gain the confidence of their children and help them to guide them with wisdom and discretion through these trying years.

A certain lady, for whose wisdom I have great respect, remarked to me that no one was ever benefited by censure, but that friendly advice and loving sympathy *only,* ever helped or inspired any one. If this be true, who is more capable to give this friendly advice and sympathy than the family physician?

Just at this point I wish to indict the legal profession for their busy methods of hunting up and exposing to the public every little violation of law by these unfortunates, for the paltry fee it affords them.

If the legal and medical professions would co-operate to help and shield the boy or girl during these irresponsible years, the world would be much better, and how many lives would be saved from infamy.

I wish just here to give an example of the splendid consequences resulting from such a humane course. There was a poor boy in our town who had been guilty of burglary. The grand jury was in session and would have found a bill against him, had it not been for the kind intercession of a young lawyer. He had known the boy from infancy; knew this to be his first offense; so asked to be allowed to go before the grand jury—present a few facts and plead the boy's cause. He knew if it ever came to trial and the boy was convicted, he would become a criminal and thereby ruined.

This young lawyer came to my office to talk the matter over and borrowed some works on adolescence. Being permitted to go before the grand jury, he told them of the boy's life, proving that he was not a criminal and ought not to be so considered, but only a boy in a very unfortunate period of life—scarcely responsible for his acts, with no true or definite ideas of property rights or money values—with just an unexplained craving for things he had not, and insufficient will power, owing to his age, to overcome temptation.

The grand jury listened with tears in their eyes and *found no bill.* Today that boy is a grown young man, sober, industrious and honest, with no shadow on his name—all due to the kindness of the lawyer friend and his willingness to *help* instead of convict.

It must be conceded that the man who is doing most for the adolescent today is Judge Ben B. Lindsay of Colorado. What a blessing to our land, if we had a Juvenile Court and a Ben B. Lindsay in every State in the Union!

In a certain small town in the Middle West there is a humane peace officer, who organized the youth of his town into a juvenile court, whose officers were elected of and by themselves. The good results from this organization are felt and appreciated by the whole citizenship. In every town or community there should be some such organization under the leadership of some good man, who is especially interested in the young.

Any organization that binds young people together on moral or religious questions, or has for its purpose mental, physical or social development, is beneficial. The Y. M. C. A. has done much for our boys and the Y. W. C. A. for our girls. The Epworth League and C. E. Societies are great factors for good. Any pledge that is taken by girls and boys and kept constantly before them tends to strengthen them in times of temptation. And particularly is this a period of temptation, for it is during these years that love becomes strongly associated with the sexual passions, awakening in the boy or girl unrequited curiosity for the opposite sex. Consequently there is a constant antagonism between the will to think and do right, and the desires of the flesh, which if left unguided and unrestrained, soon develop into lechery. This is the reason that religious organizations of all kinds are doing such great work, for "between religion and love God and nature have wrought an indissoluble bond so that neither can attain normality without the other."

The home life of the boy and girl at this particular time should be especially attractive. Parents should try to be as conservative, patient and lenient as possible, for adolescence rebels against restraint, and repulsions develop against home and school ties, resulting in truancy and vagrancy.

Especially is this true in cities and towns where the youth has no opportunity to commune with nature—nature that brings out the strongest and best in him, teaching him to find, "tongues in trees, sermons in stones, books in the running brooks and good in everything." In this generation as in every other, the men who live closest to nature are men of resolution—men who have great strength of soul and firmness of purpose—who have the power to resist temptation,

> "To leave the vain low strife
> That makes men mad—
> The tug for wealth and power—
> The passions and the cares that wither life
> And waste the little hour."

Co-operation of parents, teachers, clergymen, lawyers and physicians is needed to cope with this the greatest of questions—adolescence. For when we have guided the youth through this period and helped him to develop a strong character for good, we have rendered the greatest possible service, not only to the individual himself, but to his associates also; thereby strengthening the citizenship of the community in which he lives, his State and his nation, benefitting his whole generation and each succeeding generation.

> "No life can be pure in its purpose
> And strong in its strife
> And all life not be purer and stronger thereby."

It is fitting at this point to touch for a moment the law of heredity. The oft quoted Biblical truths, "that the fathers have eaten sour grapes and the children's teeth are set on edge," and, "the sins of the fathers are visited on the children to the third and fourth generations," are laws that we as a profession understand only too well the significance of. It is our *especial* duty as advisers to impress upon the youth the dangers of intemperance and the incurability of all venereal diseases; teaching him that not only is the individual himself weakened and his vital forces impaired, but that the results of his sins and indiscretions are transmitted to his offspring, often times victimizing them as delinquents, defectives or degenerates. We cannot be too much concerned on this subject, and we are *particeps criminis* if we neglect to repeatedly impress upon them these grave consequences.

We should establish such a bond of sympathy and confidence between ourselves and the youth of our clientele that they may feel free to come to us at all times for instruction and advice.

By this rational method it would be possible to produce a race well born and well bred. For, if each generation as it is evolved would universally adopt eugenic principles, improving and culturing its offspring to the utmost capacity, then would the defects and weakness of the human race be gradually eliminated, until it would attain again that perfection which it had in the beginning—the image and likeness of God.

Weatherford, Tex., Oct. 13, 1913.

